# STAT3 exerts pro-tumor and anti-autophagy roles in cervical cancer

**DOI:** 10.1186/s13000-021-01182-4

**Published:** 2022-01-20

**Authors:** Lin Wu, Bowen Shen, Junpeng Li, Huirong Zhang, Ke Zhang, Yao Yang, Zhenyu Zu, Dongxiang Shen, Min Luo

**Affiliations:** 1 The First School of Clinical Medicine, Southern Medical University, Guangzhou, China; 2 Department of Obstetrics and Gynecology, General Hospital of Southern Theatre Command of PLA, Guangzhou, China; 3 Department of Clinical Medicine, Zhongshan Medical College of Sun Yat-sen University, Guangzhou, China; 4 Department of Gynecology, the Second Affiliated Hospital of Guangzhou University of Chinese Medicine, Guangzhou, China; 5 Department of Obstetrics and Gynecology, the third affiliated hospital of Guangzhou medical university, Guangzhou, China; 6 Department of Medical Engineering, General Hospital of Southern Theatre Command of PLA, Guangzhou, China

**Keywords:** Cervical cancer, Autophagy, STAT3, LC3B

## Abstract

**Background:**

STAT3 plays an important role in cervical cancer. LC3B, the most potential molecular biomarker of autophagy that may promote or inhibit cancer progression, can be downregulated by STAT3. However the role of STAT3 in the autophagy of cervical cancer remains unclear.

**Purpose:**

This study aimed to evaluate the relationship between STAT3 and LC3B in protein level, and verify whether STAT3 promotes proliferation, migration and plate colony formation by inhibiting autophagy of cervical cancer cells through bcl2-beclin1 axis.

**Results:**

STAT3 was overexpressed in cervical cancer tissues, and negatively correlated with the expression level of LC3B. STAT3 knockout or knockdown significantly increased the autophagy level and decreased proliferation, migration, plate colony formation and subcutaneous tumorigenesis of cervical cancer cells in vitro and in vivo. STAT3 is known to mediate autophagy through Bcl2-Beclin1 complex. Bcl2 was positively whereas Beclin1 negatively correlated with STAT3 expression, indicating that Bcl2-Beclin1 complex involved in this transition.

**Conclusion:**

STAT3 may upregulate the autophagy level of cervical cancer cells through the Bcl2-Beclin1 axis. This indicates that STAT3 may be an important prognostic and therapeutic target for cervical cancer.

## Introduction

Cervical cancer is the second leading cause of cancer-related death among women in developing countries, and has a high recurrence rate and drug resistance [1]. Although the incidence of cervical cancer may be controlled with the application of HPV vaccines, the incidence and mortality of cervical cancer remain high especially in developing countries. Therefore, studies on the pathophysiological mechanism of cervical cancer are still necessary.

Signal transducer and activator of transcription 3 (STAT3) is an important regulator in malignant tumor [2], which also plays an essential role in the HPV positive cervical disease [2, 3]. Numerous studies report [4–6] that STAT3 could affect autophagy through modifying the key molecular such as LC3B and then affect the progression of the tumor. There were studies [7–9] implied that autophagy is closely related to the proliferation, differentiation and metastasis of numerous cancer cells. STAT3 in the nucleus could be the main transcriptional enhancer of several genes, such as BCL2, BECN1, PIK3C3, CTSB, CTSL, that inhibit or activate cell autophagy. The anti-autophagy function mainly lies in that it can hinder the formation of BECN1/PIK3C3 complex [10]. The reason is that Beclin1, the key protein of autophagy initiation, contains a BH3 domain, which may interact with the anti-apoptotic BCL2 family activated by STAT3 [4]. However, how STAT3 regulates the autophagy of cervical cancer cells in cervical cancer remains unclear.

Recently, studies [2, 11, 12] have shown that STAT3 has a significant impact on the proliferation and apoptosis of cervical cancer cells, and mainly plays the role as one of the key oncogenes, promotes the proliferation of cervical cancer cells and inhibits their apoptosis. At the same time, local recurrence, distant metastasis and drug resistance of postoperative cervical cancer become difficulties in the treatment of cervical cancer [13]. The study of autophagy in cervical cancer provides a new strategy for the treatment of cervical cancer based on the function of autophagy to improve the resistance to “harsh environment” of cancer cells. However, the relationship between STAT3 and autophagy as an important pathway in regulating the physiology and pathology of cervical cancer cells has not been elucidated. This paper will further explore the correlation between STAT3 and LC3B in cervical cancer tissues, and the proliferation, metastasis and autophagy of cervical cancer cells after regulating the activity of STAT3 gene. This serves to clarify the regulatory mechanism between STAT3 and autophagy and its effect on the proliferation and metastasis of cervical cancer cells. It will provide new ideas for the treatment and prognosis of cervical cancer, especially advanced and recurrent cervical cancer.

## Methods and materials

### Ethics statement

A total of 46 primary cervical cancer tissues from cervical carcinoma patients were collected at the Department of Pathology in General Hospital of Southern Theatre Command of PLA during the period from July 2015 to March 2019. All specimens were immediately frozen in liquid nitrogen and stored at − 80 °C until use. This study was approved by the ethical committee of General Hospital of Southern Theatre Command of PLA and was conducted according to the Helsinki declaration. Written informed consent was obtained from all patients according to the guidelines approved by the institutional research board. All animal experiments were conducted following appropriate guidelines.

### Immunohistochemistry

Immunohistochemistry was performed to detect STAT3 and LC3B protein expression in cancer tissue samples. The method refers to the previous research of this group [14].

### Cell culture and main regents

Human cervical cancer cell line (SiHa and HeLa) were obtained from the Cell Bank, Type Culture Collection, Chinese Academy of Sciences (CBTCCCAS). The methods of cell culturing was conducted according to our previous research [14]. Then, human STAT3 sgRNA and shRNA targeted STAT3 were designed, the corresponding vector was constructed, lentiviral expression vector was used to transfect SiHa and Hela cells to construct STAT3-knockout (STAT3-KO) or STAT3-knocdown (sh-STAT3) cell lines, respectively. Finally, the transfection efficiency after transfection was determined by Western blot. Chloroquine phosphate (PHR1258) was purchased from Sigma-Aldrich; antibodies of stat3 (10253–2-AP), Bcl2 (60178–1-Ig), Beclin1 (11306–1-AP), P62 (18420–1-AP) were purchased from Proteintech. LC3B (3868S), β-actin (3700S) antibodies were purchased from CST (Danvers, MA, USA).

### Knockout STAT3 of cervical cancer SiHa cells by CRISPR/Cas9

Design of sgRNA: CAGTGGCTGCAGTCTGTAGA for the exons of human STAT3 gene, and clone it into a lentiviral expression vector (lentiCRISPRv2). The vector requires simultaneous expression of the target sgRNA and Cas9 protein; the cell selection resistance is puromycin. First, 4 μg plasmids and lentiviral expression vector were cotransfected to the 293 T cells, and 30 μl lipofectamine™ 2000 was added. Next,10 ml of fresh medium was added after 6 h, followed by filtering the culture supernatant using 0.45 μm non-nitrocellulose Filter (cellulose acetate and other filters) after 48 h to sterilize and to remove cell debris and contaminated packaging cells. The mixture was stored temporarily at 4 °C, and stored at − 80 °C for long term use. Subsequently, 10 ml of virus-containing supernatant with 8 μg/ml Polybrene (prepared to 1000× working solution) were added to the medium of SiHa cells; the normal medium was replaced with 10% FBS DMEM after 6 h; the concentration of 0.5μg /ml puro screening was used after 24 h; the medium was changed every 1–2 days (all contain the same puro concentration, the puro concentration was adjusted according to the cell death); stable cell lines was selected after about 2 weeks.

### Cell function assays in vitro

CCK8 assay was performed to detect cell viability. After digesting adherent SiHa cells into cell suspension, 1 × 10^3^ cells were seeded in a well of 96-well plates, where each group had four duplicate wells. After SiHa cells attaching to the plate, 10 μL of CCK-8 was added to each well. Optical density was measured at 450 nm, 2 h after CCK-8 was added. The same operation was performed for 7 days continuously. Cell migration was analyzed by transvell assay, which could show the migrated cells by 0.5% crystal violet through microscope (magnificence 200X). Colony formation assay was that 300 cells were seeded in 6 well plates for 14 days, and then each well was stained with 0.5% crystal violet for 30 min at room temperature. All assays above were performed in triplicates.

### Western blotting

Protein extraction was performed using RIPA reagent. Lysis protein was collected and NanoDrop2000 system (Thermo Scientific, USA) was adopted to adjust protein concentrations. Primary antibodies were incubated, shaken at 4 °C overnight, and secondary antibodies were incubated at room temperature for 1 h. Images were collected using the Imaging System (Tanon 5200 Multi) with ECL and analyzed by ImageJ.

### Observation of autophagosomes and autophagolysosomes using confocal microscope and electron microscope

First, about 1000 cells were seeded in Confocal small dishes. After 24 h, DMEM/F-12 with or without 50μmoL/L chloroquine phosphate was added. Next, each dish was added with 1.0 μmoL/L DALGreen and DAPRed Working Solution. Then autophagosomes and autophagolysosomes were observed with confocal microscope after 24 h. The test was performed in triplicate. Blue ZEN and ImageJ software were used to analyze the results. Electron microscope was used to detect the autophagosomes and autophagolysosomes of cells according to manufacturer’s instructions.

### Animal experiments

Four four weeks old female nude mice were maintained in the SPF-grade animal laboratory, acquired from Guangdong Medical Laboratory Animal Center (Guangdong, China). All procedures were approved by the Animal Research Ethics Committee of General Hospital of Southern Theatre Command of PLA. 5 × 10^6^ cells were inoculated subcutaneously in the groin for 5 weeks. Tumor volume was measured and recorded every week. Lastly, tumors were cut off and weighed for analysis.

### Statistical analysis

All data were analyzed statistically via a t test, one-way ANOVA or Pearson Correlation Analysis by using IBM SPSS Statistic (version 22) or Graph Prism7, with *P* < 0.05 as significant level. Results were expressed as the mean ± SD or frequency from three or more repetitions assays.

## Results

### STAT3 is highly expressed in cervical cancer patients and is negatively correlated with LC3B

**Fig. 1:**
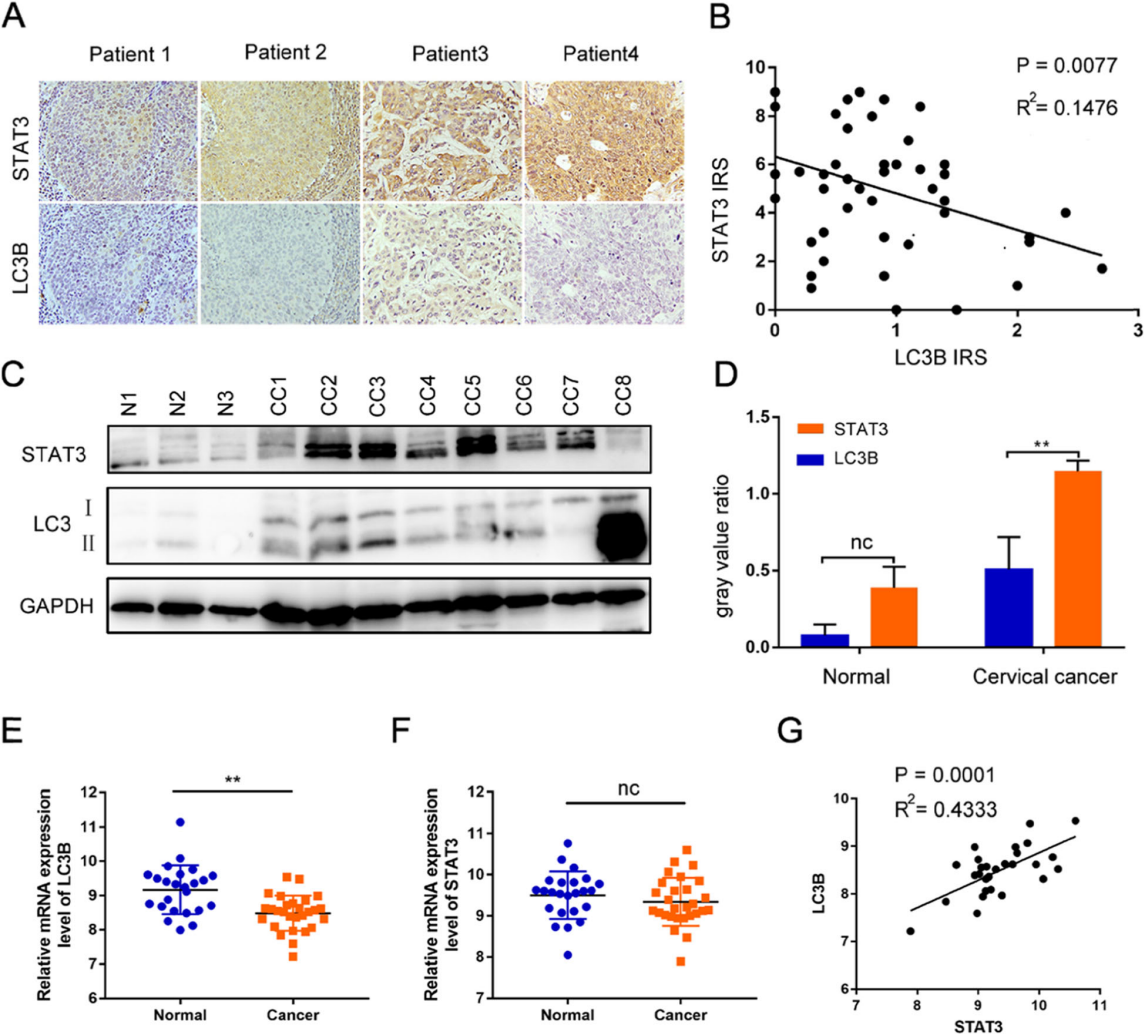
STAT3 is highly expressed in cervical cancer patients and is negatively correlated with LC3B.**A**. the expressions of STAT3 and LC3B in cervical cancer patients; **B**. The STAT3 immunoreactive score (IRS) is negatively related to LC3B IRS; **C** and **D**. P-STAT3 and LC3B were detected by western blotting, then analyzed by Image J; **E**. The mRNA expression of LC3B in normal cervical tissue was significantly higher than that in cervical cancer by GDS3233.**F**. The mRNA expression of STAT3 in normal cervical tissue was no different with that in cervical cancer. **G**. STAT3 correlates positively with LC3B at the mRNA level. (“ns” no significance, “ * ” *p*<0.05, “ ** ” *p*<0.001)

In order to explore the role of STAT3 in the progression of cervical cancer, we first analyzed the relationship between the expression of STAT3 and LC3B. We found that STAT3 is negatively related to LC3B in 46 cervical cancer patients (Fig. 1 A-D). At the same time, the expression of STAT3 or LC3B in patients with different histological type, differentiation degree, FIGO stage, tumor size, lymph node metastasis and vascular infiltration of cervical cancer was detected by immunohistochemistry. Results showed that the positive expression rate of STAT3 in advanced cervical cancer is higher than that in early cervical cancer (Table 1). These results were in agreement with the results of the GEO database, suggesting that the mRNA level of normal cervix is significantly higher than that of cervical cancer detected by microarrays (Fig. 1 E). However, the mRNA expression level of STAT3 between primary cervical cancer and normal tissue is not significantly different (Fig. 1F), and STAT3 correlates positively with LC3B at the mRNA level, which differs from the correlation result at the protein and tissue level (Fig. 1G), which indicates that the regulation of STAT3 in autophagy may be complex in cervical cancer.

**Table 1:** The correlation between STAT3 and clinicopathological characteristics

Clinicopathological factors	n	Positive	Negative	*χ*^*2*^	*p*
**Age (years)**					
< 45	18	14	4	–	0.613^a^
≥ 45	28	22	6		
**Histological type**					
Squamous	40	33	7	–	0.107^a^
Noun-squamous	6	3	3		
**Tumor differentiation degree**					
Low	31	25	6	–	0.706^a^
Noun-low	15	11	4		
**FIGO Stage**					
IA-IB	21	13	8	4.271	0.039
IIA-IVB	25	22	3		
**Tumor size (cm)**					
≤ 4	30	21	9	–	0.064^a^
> 4	16	15	1		
**Lymph node metastasis**					
YES	8	4	4	–	0.055^a^
NO	38	32	6		
**Vascular infiltration**					
YES	10	8	2	–	0.553^a^
NO	36	27	9		

*FIGO* Federation International of Gynecology and Obstetrics

^a^The statistical method is Fisher test

### STAT3 promotes proliferation, migration and clonal formation of cervical cancer cells in vitro and in vivo

**Fig. 2:**
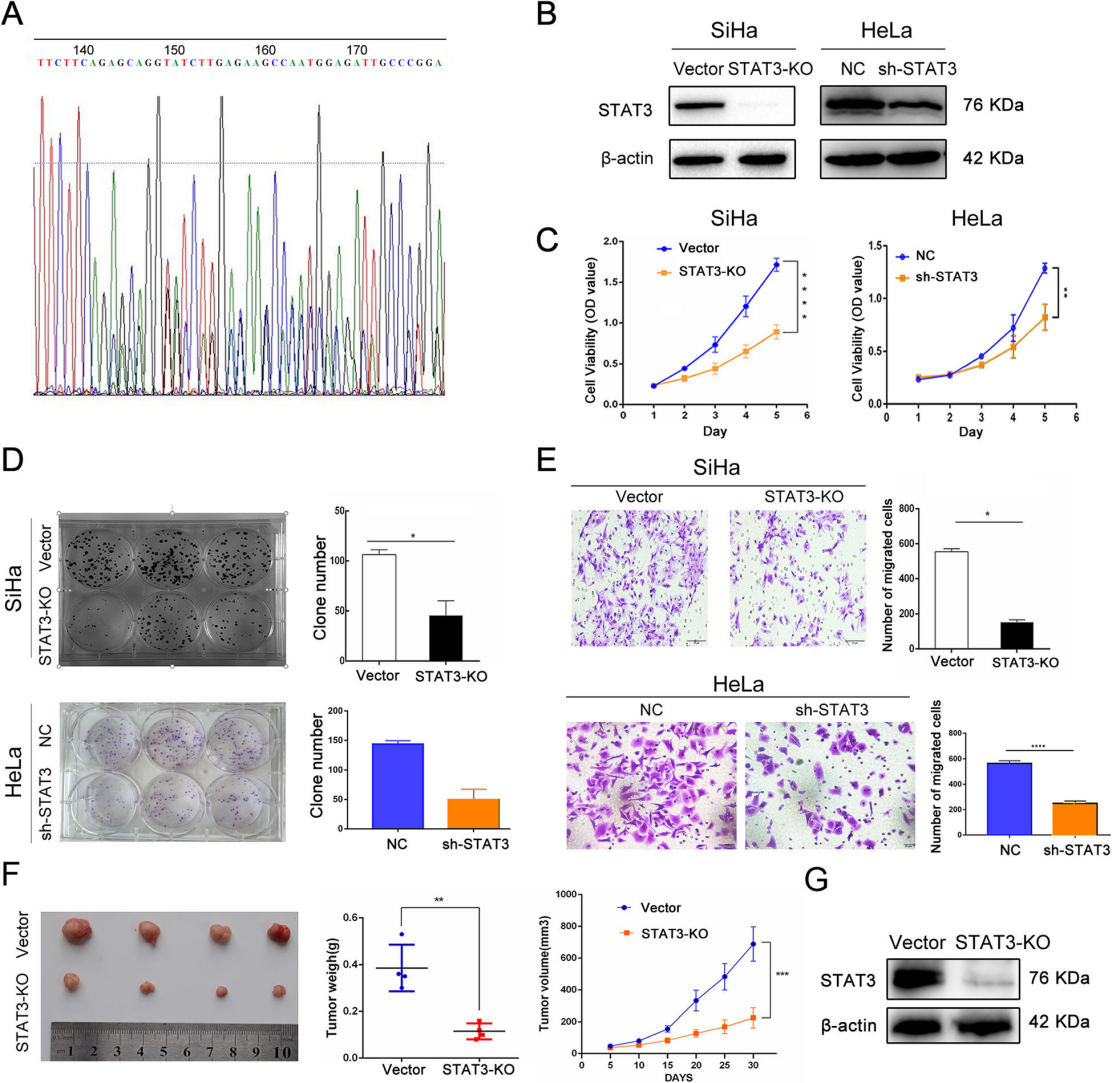
STAT3 promotes proliferation, migration and clonal formation of cervical cancer cells. **A** STAT3 guide-RNA sequence; **B** The knockout and knockdown efficiency was verified by western blotting; **C**-**E**. cck8, clone formation, transwell assays detected the proliferation ability, clone formation and migration ability; **F** Representative appearance of subcutaneous tumors, the tumor weight and the tumor growth curve in the study; **G** The expression of STAT3 in tumor tissues was detected by western blotting. (“ns” no significance, “ * ” *p*<0.05, “ ** ” *p*<0.01, “ *** ” *p*<0.001, “ **** ” *p*<0.0001)

To verify the role of STAT3 in cervical cancer cells, we first used CRISPR-CAS9 technique to knock out STAT3 gene in SiHa cells, and verified its efficiency by western blotting. Results showed that CRISPR-CAS9 successfully knocked out the STAT3 gene fragment of SiHa cells (Fig. 2A, B). Additionally, sh-NC/−STAT3 Hela cell line were also constructed and the transfection efficiency was determined by Western blot (Fig. 2B). CCK8 assay indicated that the proliferation ability of SiHa and Hela cells was significantly lower than that of the control cells after knockout or knockdown of STAT3 (Fig. 2C); clone formation assay implied that the clone formation of SiHa and Hela cells after knockout or knockdown of STAT3 was significantly lower than that of control cells (Fig. 2D). Transwell assay demonstrated that the migration ability of SiHa and Hela cells was significantly lower than that of control cells after knockout or knockdown of STAT3 (Fig. 2E). Therefore, a series of in vitro experiments have proven that STAT3 plays an indispensable role in the proliferation, metastasis and colony formation of cervical cancer cells.

After the in vitro experiment to verify the effect of STAT3 on the proliferation and colony formation of cervical cancer cells, in vivo experiment was carried out in order to be more convincing. The experiment of subcutaneously transplanted tumor in nude mice showed that the tumorigenesis ability of SiHa cells after knockout STAT3 was significantly weaker than that of control cells (Fig. 2F), and the expression of STAT3 in tumor tissue of nude mice detected by western blotting was consistent with that of tumorigenesis (Fig. 2G). Based on the above results in vitro and in vivo, STAT3 plays an active role in the proliferation, migration, colony formation and subcutaneous-transplant tumor formation of cervical cancer cells.

### STAT3 inhibits the autophagy level of cervical cancer cells

**Fig. 3:**
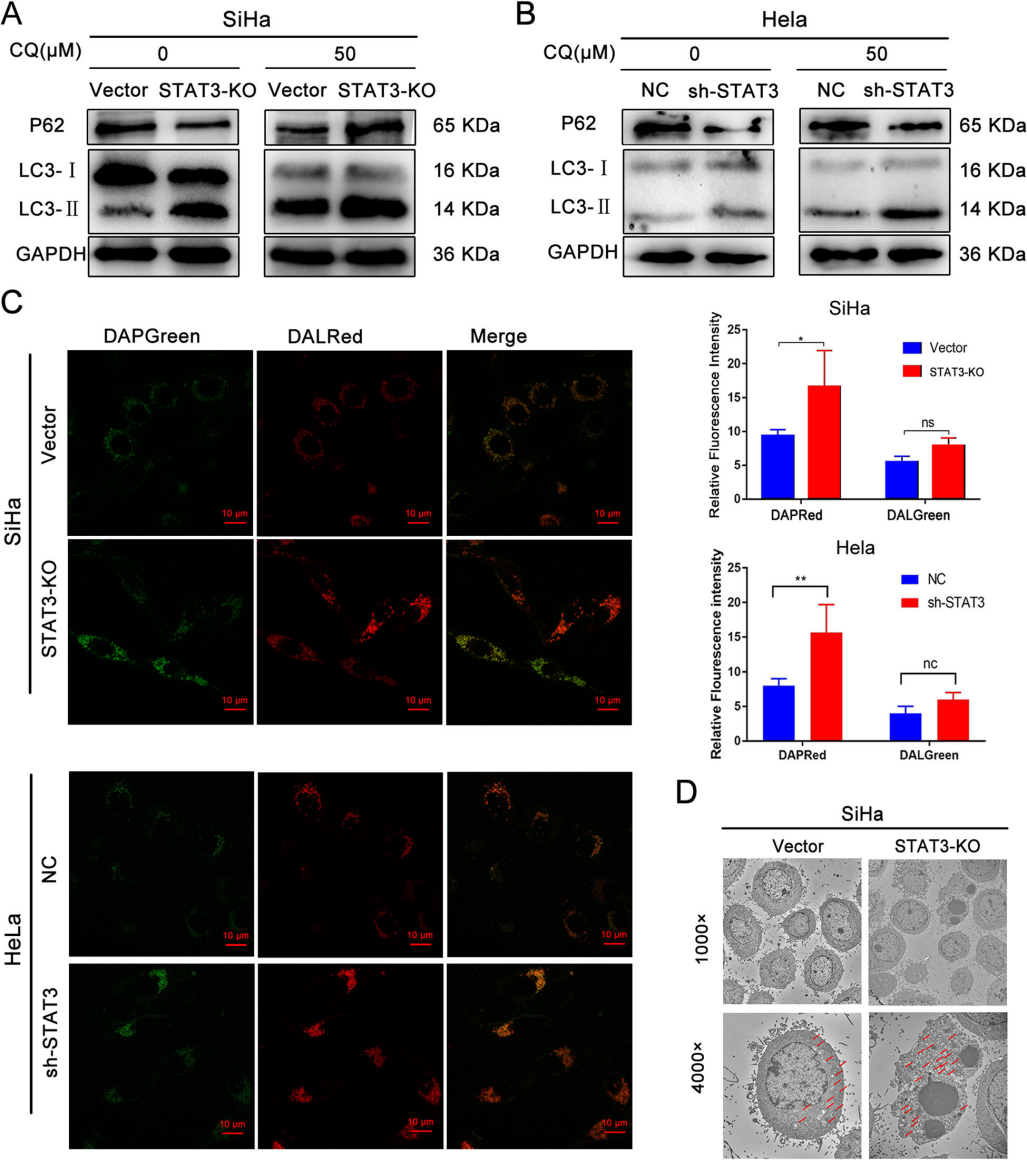
STAT3 inhibits autophagy level of cervical cancer cells. **A** and **B** The autophagy marker proteins in cervical cancer cells treated with or without chloroquine were detected by western blotting; **C** The autophagosomes and autolysosomes were observed under Inverted Confocal Fluorescence Microscope; Semi-quantitative fluorescence of “C”. **D**. The autophagosomes were observed by electron microscope (red arrow: autophagosomes or autolysosomes); (“ns” no significance, “ * ” *p*<0.05)

Prior studies proved that STAT3 promotes the proliferation and migration abilities of cervical cancer cells, and then whether STAT3 influence the autophagy of cervical cancer cell remains unclear. Hence, in order to explore the relationship between STAT3 and autophagy of cervical cancer cells, the expression levels of autophagy marker proteins (Beclin1, P62, LC3B) in STAT3-KO/sh-STAT3 and relative control cells treated with or without chloroquine were detected by western blotting. The higher the expression levels of beclin1 and LC3B or the lower the expression level of p62, indicating that the higher the level of autophagy [15]. Results showed that the expression levels of beclin1 and LC3B increased, while the expression level of p62 decreased in STAT3-KO/sh-STAT3 cells (Fig. 3A). To show the changes in autophagy level of cervical cancer cells more vividly, we further applied Microscope technology. Firstly, observation using Electron microscope and Inverted Confocal Fluorescence Microscope both showed that the levels of autophagosomes and autophagy lysosomes in STAT3-KO/sh-STAT3 cells were higher than those in vector/NC cells (Fig. 3B-D). In short, STAT3 not only promotes the abilities of proliferation and migration in cervical cancer cells, but also restrain the autophagy of cervical cancer.

### STAT3 regulates autophagy of cervical cancer through bcl2-beclin1 axis

**Fig. 4:**
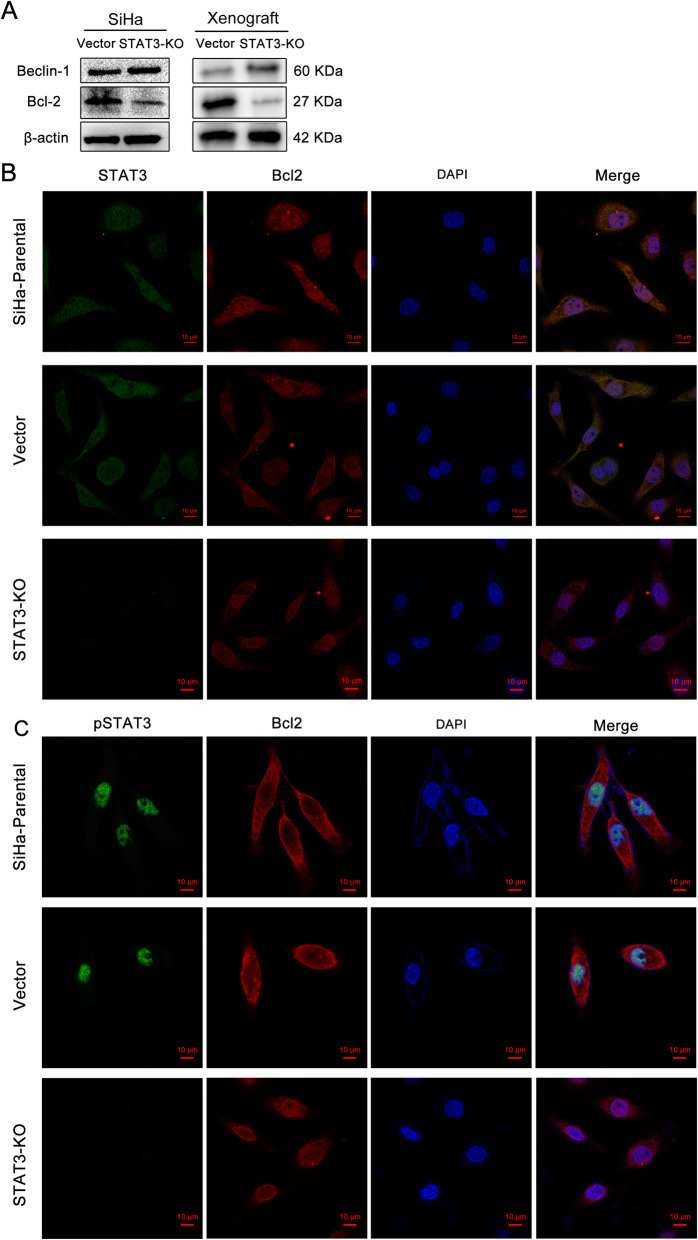
STAT3 regulates autophagy of cervical cancer through bcl2-beclin1 axis. **A** The protein expression of Beclin1, Bcl2 in cervical cancer cells and the Xenograft; **B** and **C**. Intracellular STAT3, PSTAT3, Bcl2 protein expression sub localization of cervical cancer

It is well-known that beclin1 is a key initiation molecule in autophagy process [16]. Bcl2 binds to beclin1 to prevent the formation of autophagy initiation complex [17]. In order to explore the specific mechanism of the effect of STAT3 on the proliferation, metastasis and autophagy of cervical cancer cells, we found that bcl2 and beclin1 may play a corresponding role in the downstream of STAT3. Results of western blotting showed that after knockout the expression of STAT3 in SiHa cells, the expression of bcl-2 protein showed a downward trend, while beclin-1 showed an upward trend (Fig. 4A). The same verification was carried out in tumor tissues of nude mice, and the results were consistent with those in cell level (Fig. 4A). Subsequently, immunofluorescence experiments were carried out on SiHa cells to localize STAT3, pSTAT3 and bcl2 (Fig. 4B, C). Results showed that STAT3 was mainly located in the cytoplasm, pSTAT3 was mainly located in the nucleus, and BCL2 was localized in both areas. These results suggest that STAT3 may affect the biological function of cervical cancer cells by affecting the bcl2-beclin1 axis. Although the immunofluorescence experimental pictures of the knockdown group and the control group showed no significant difference in its location, there was a significant difference in its content, which could be obtained from the WB results.

## Discussion

STAT3 is an important transcription factor, which mediates extracellular signals by interacting with polypeptide receptors on the cell surface. STAT3 protein is activated at the transcriptional level mainly through tyrosine phosphorylation. The activated STAT3 dimer trans-locates to the nucleus and binds to sequence-specific DNA elements to transcribe the target gene [18]. STAT3 plays the role of oncogene in most malignant tumors [19–26]. However, the role of STAT3 in cervical cancer is not clear. This study found that STAT3 is highly expressed in cervical cancer tissues, has a negative correlation with LC3B, one of the most essential autophagy molecular, and the results of this study are consistent with the results shown in GEO database. In vivo and in vitro experiments have found that STAT3 and autophagy are closely related to cell oncogenesis ability. Next, this study verified that STAT3 promotes the proliferation, metastasis, clone formation and subcutaneous tumorigenesis of cervical cancer cells in vivo and in vitro. The possibility of STAT3 as a targeted therapeutic molecular for cervical cancer is discussed in this study.

Recently, an increasing number of studies demonstrated that STAT3 can also regulate autophagy which involved in many steps, from the assembly of autophagosomes to their maturation [4]. STAT3 in the nucleus could be the main transcriptional enhancer of several genes, such as BCL2, BECN1, PIK3C3, CTSB, CTSL that inhibit or activate cell autophagy. The anti-autophagy function mainly lies in that it can hinder the formation of BECN1/PIK3C3 complex [10]. So, in cervical cancer, does STAT3 affects autophagy level of cervical cancer cells, and in which way it affects the autophagy level of cervical cancer cells? In this study, we found that the level of autophagy increased significantly after STAT3 knockout or knockdown in cervical cancer cells by detecting the protein expression levels of Bcl-2 and Beclin-1, it was found that the expression of Bcl-2 decreased, while the expression of Beclin-1 increased. It was confirmed that the autophagy level of cervical cancer cells increased after the downregulation of STAT3, which may play a role through the Bcl2-Beclin1 axis. In our study, STAT3 correlates positively with LC3B at the mRNA level, which differs from the correlation result at the protein and tissue level. However, correlation is not causation. Based on our experimentation and statistical analysis, STAT3 is a negative effector of LC3B - a classic marker of autophagy, and probably regulate its expression via proteins interaction.

It is found that autophagy plays opposing, context-dependent role in cancer. Autophagy may provide tumor cells with nutrients for survival by decomposing intracellular nutrients and play certain roles in drug resistance [27]; on the other hand, excessive autophagy may also lead to cell death [28]. So far, autophagy inhibition or stimulation have been proven to be an effective supplement to several cytotoxic agents and targeted therapeutic drugs in a variety of preclinical models [29–31]. Therefore, whether patients suffering from cervical cancer could benefit from autophagy inhibiting agents still needs for further discussion. According to our findings, after impeding genic STAT3, the productivity and metastasis of cervical cancer cells decreased, while the autophagy increased. If combining with autophagy inhibitor, it will act either synergistic action or counter action, which directly stimulates or inhibits autophagy in cancer therapy. Therefore, appropriate and effective selection of autophagy-targeted interventions combined with other treatments to improve the prognosis of refractory cervical cancer is still a challenge for further research.

## Data Availability

Data are available on reasonable request from the corresponding author due to privacy or other restrictions.
